# Knowledge and Perceptions of Highly Pathogenic Avian Influenza (HPAI) among Poultry Traders in Live Bird Markets in Bali and Lombok, Indonesia

**DOI:** 10.1371/journal.pone.0139917

**Published:** 2015-10-02

**Authors:** Johanna Kurscheid, Joanne Millar, Muktasam Abdurrahman, I Gusti Agung Ayu Ambarawati, Wayan Suadnya, Ria Puspa Yusuf, Stanley Fenwick, Jenny-Ann L. M. L Toribio

**Affiliations:** 1 School of Veterinary and Life Sciences, Murdoch University, Murdoch, Western Australia, Australia; 2 School of Environmental Sciences, Faculty of Science, Charles Sturt University, Albury, New South Wales, Australia; 3 Research Center for Rural Development. Mataram University, Mataram, Indonesia; 4 Agribusiness Study Program, Faculty of Agriculture, Udayana University, Denpasar, Bali, Indonesia; 5 Department of Infectious Disease and Global Health, Cummings School of Veterinary Medicine, Tufts University, Boston, Massachusetts, United States of America; 6 Faculty of Veterinary Science, Camden Campus, The University of Sydney, Camden, New South Wales, Australia; University of Hong Kong, CHINA

## Abstract

Highly Pathogenic Avian Influenza (HPAI) has been prevalent in Indonesia since 2003 causing major losses to poultry production and human deaths. Live bird markets are considered high risk areas due to the density of large numbers of mixed poultry species of unknown disease status. Understanding trader knowledge and perceptions of HPAI and biosecurity is critical to reducing transmission risk and controlling the disease. An interview-administered survey was conducted at 17 live bird markets on the islands of Bali and Lombok in 2008 and 2009. A total of 413 live poultry traders were interviewed. Respondents were mostly male (89%) with a mean age of 45 years (range: 19–81). The main source of AI information was TV (78%), although personal communication was also identified to be an important source, particularly among female traders (60%) and respondents from Bali (43%). More than half (58%) of live poultry traders interviewed knew that infected birds can transmit HPAI viruses but were generally unaware that viruses can be introduced to markets by fomites. Cleaning cages and disposing of sick and dead birds were recognized as the most important steps to prevent the spread of disease by respondents. Two thirds (n = 277) of respondents were unwilling to report sudden or suspicious bird deaths to authorities. Bali vendors perceive biosecurity to be of higher importance than Lombok vendors and are more willing to improve biosecurity within markets than traders in Lombok. Collectors and traders selling large numbers (>214) of poultry, or selling both chickens and ducks, have better knowledge of HPAI transmission and prevention than vendors or traders selling smaller quantities or only one species of poultry. Education was strongly associated with better knowledge but did not influence positive reporting behavior. Our study reveals that most live poultry traders have limited knowledge of HPAI transmission and prevention and are generally reluctant to report bird deaths. Greater efforts are needed to engage local government, market managers and traders in education and awareness programs, regulatory measures and incentive mechanisms. Understanding and evaluating the social responses to such an integrated approach could lead to more effective HPAI prevention and control.

## Introduction

Since the first outbreaks in 2003, highly pathogenic avian influenza (HPAI) H5N1 has spread rapidly and is now endemic in poultry in most provinces in Indonesia [1], causing significant social and economic impacts on poultry producers and the industry [2–5]. The human mortality rate from HPAI H5N1 in Indonesia is the highest in the world. Between 2003 and May 2015, 199 laboratory confirmed cases of infection in humans have occurred of which 165 have been fatal, mostly on Java but also on the islands of Sumatra, Sulawesi, Bali and Lombok [6]. The spread of HPAI in Indonesia continues, most likely via movement of infected poultry, despite control programs focused on movement controls, culling and to a lesser degree vaccination [7–9].

Morris and Jackson [10] identified several factors facilitating the spread of HPAI virus in Asia, either directly or indirectly. Firstly, high-risk farming and handling practices such as raising poultry of mixed species or in a free-range type setting in rural or urban areas. Secondly, unsafe transport of live birds via infected vehicles and bird cages and thirdly, a lack of biosecurity measures at live bird markets (LBMs). Live bird markets receive and distribute large numbers of mainly uninspected birds of unknown infectious status [11–13]. Poultry species tend to be mixed in the same cages, which may lead to cross infection [8, 14]. Studies have also shown that biosecurity measures, such as separating sick birds and disinfecting equipment and vending areas are often inadequate at live bird markets [8, 9, 15, 16].

The role of poultry traders, including collectors and vendors, is crucial to increasing biosecurity standards at poultry markets and reducing the persistence and circulation of avian influenza viruses [17–19]. Traders are at risk of infection due to daily contact with birds, and they can unwittingly transmit the virus from market to market or to and from farms when transporting live birds [18, 20]. There has been limited research on the knowledge and practices of poultry traders compared to poultry farm workers.

In 2009, a survey of 140 Nigerian male poultry traders and market workers was carried out at three traditional live bird markets. The study found that knowledge of certain key aspects of HPAI transmission (e.g. poultry and wild birds are vectors of the disease) was very high among respondents but awareness of other aspects (e.g. HPAI can be transmitted to people by handling uncooked poultry) was very limited [20]. Research conducted among 352 traditional poultry market workers and shoppers in the same year in Taiwan (where there had been several outbreaks of H5N2, but not H5N1) found that recommended AI preventative behavior (e.g. wearing a face mask and washing hands after any contact with poultry) was highly correlated with having school or university education and also correlated with correct knowledge of bird fatality rates, severe cases and local outbreaks [21]. The following year, Manabe et al. [22] reported the findings of a survey conducted in Vietnam of 543 respondents residing either in a community which had experienced H5N1 outbreaks (which was also in a more rural setting) or in one which had not (a more urban setting). They found that knowledge of H5N1 and preventative measures was influenced by education and awareness of local outbreaks, as well as occupation and economic conditions.

HPAI knowledge of poultry farm workers in Italy, Nigeria and China were also found to be greater with higher levels of education and among those who believed they were at high risk of contracting HPAI [23–25]. In addition, there appears to be higher awareness levels of HPAI amongst urban or peri-urban poultry workers and consumers than rural counterparts [24, 26]. These findings are not surprising given the low education levels of poultry workers and traders, lack of adequate facilities in some countries and the lack of involvement of poultry workers or traders in disease surveillance and control, which are normally carried out by government services [27–29].

The influence of HPAI information sources and education programs (e.g. mass media, training and community surveillance programs) on poultry workers’ or villagers’ knowledge have been investigated in some countries [25, 26, 28, 30, 31]. Television was the main source of HPAI information in Nigeria, Laos and Vietnam [24, 26, 30], while radio was more important in Nepal [31]. Involvement of local healthcare workers and administrators in Vietnam in HPAI H5N1 education and outreach was found to be highly influential in increasing HPAI awareness and building community trust in using health services [30].

Sims [19] highlighted the enormous investment that has gone into “behavior change communication” in countries where HPAI infection is endemic. Whilst HPAI knowledge has increased, it has not led to universal changes in HPAI preventative behavior. Understanding and addressing the more subtle social and cultural drivers of behavioral change is necessary [8, 19].

The objectives of this study were to gain insight into poultry trader knowledge and perceptions towards HPAI, reporting and biosecurity in live bird markets (LBMs) in Bali and Lombok. Furthermore, we aimed to identify whether differences in knowledge and perceptions exist between different socio-demographic profiles or trader characteristics, and to determine whether the type or diversity of HPAI information sources to which respondents had been exposed influenced outcomes. This information is valuable for developing and improving current approaches to address behavior change among poultry traders and to minimize the risks associated with HPAI infections in both poultry and people.

## Methods

The study protocol was reviewed and approved by the Murdoch University Human Research Ethics Committee, Perth, Western Australia (Permit number 2008/162). Participants were verbally provided with information of the study objectives, purpose and format, and assured of their anonymity. Written consent was obtained by all participants prior to the commencement of the interview.

### Selection of markets and locations

**Fig 1:**
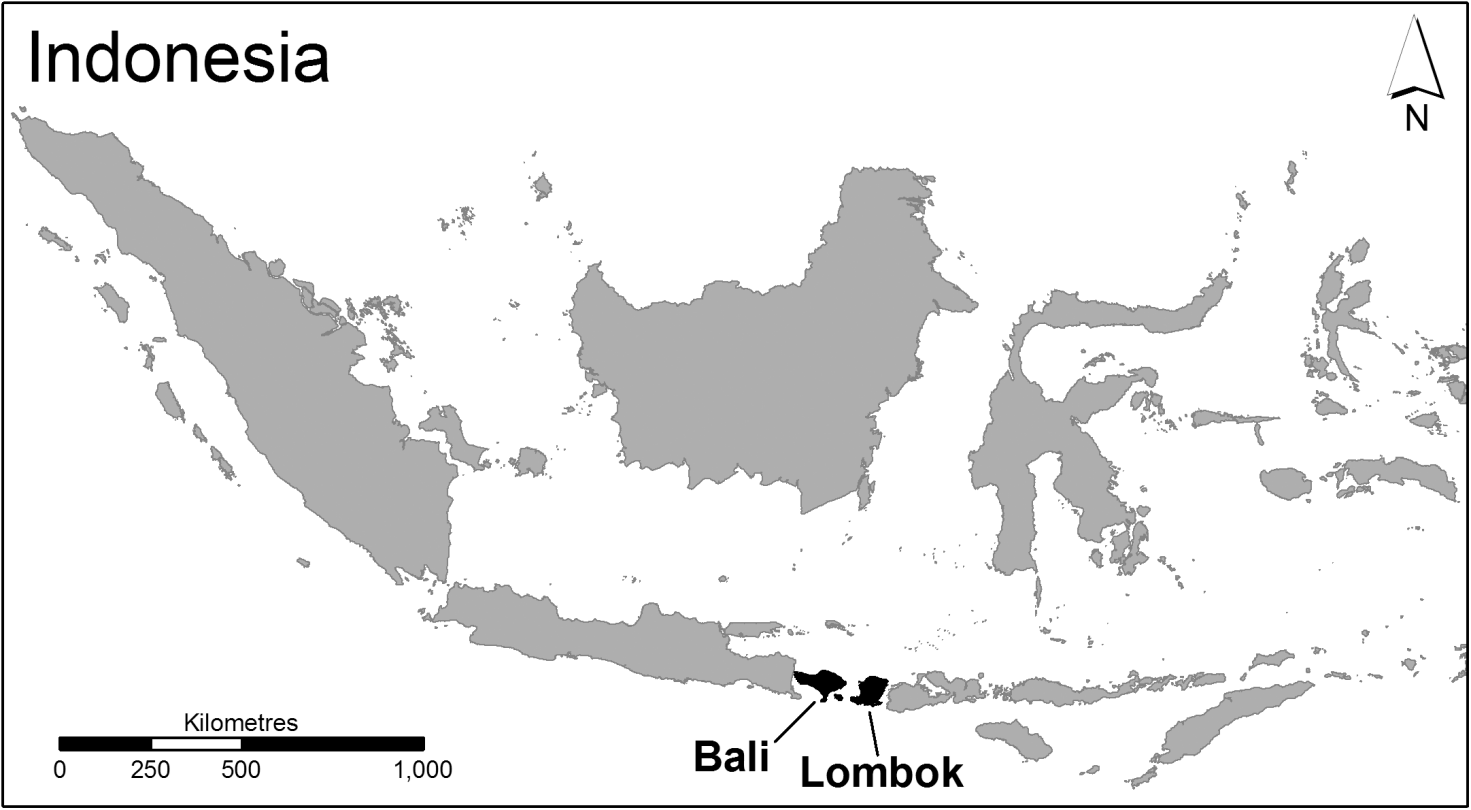
Location of Bali and Lombok in Indonesia (Source: Charles Sturt University).

The study was carried out on the islands of Bali and Lombok in Indonesia (Fig 1). These islands were selected based on their location, similar poultry industries and differing HPAI H5N1 outbreak status at the time of the study. The poultry industry on both islands consists of Sector 3 commercial farms (layers and broilers) and Sector 4 backyard farms (village chickens) [32], which are both at greater risk of HPAI infection than large-scale, industrialized poultry farms (i.e. Sector 1 or 2 farms) due to low levels of biosecurity [33]. Bali’s close proximity to Java, which is believed to be the epicenter of the HPAI H5N1 infection in Indonesia [10], places it in a particularly vulnerable position as poultry moves through illegal channels between these islands [16, 34]. At the commencement of this study Lombok had no laboratory confirmed human HPAI H5N1 fatalities and although sporadic poultry outbreaks had been reported and confirmed locally [32] it was not considered to be endemic on the island. In contrast human fatalities had occurred in Bali and the island province was considered to be HPAI H5N1 endemic [35]. Official reports of HPAI H5N1 poultry outbreaks in Lombok began to surface in late 2011 as FAO Participatory Disease Surveillance and Response (PDSR) data became available and the first human fatality was reported in March, 2012 [36].

**Fig 2:**
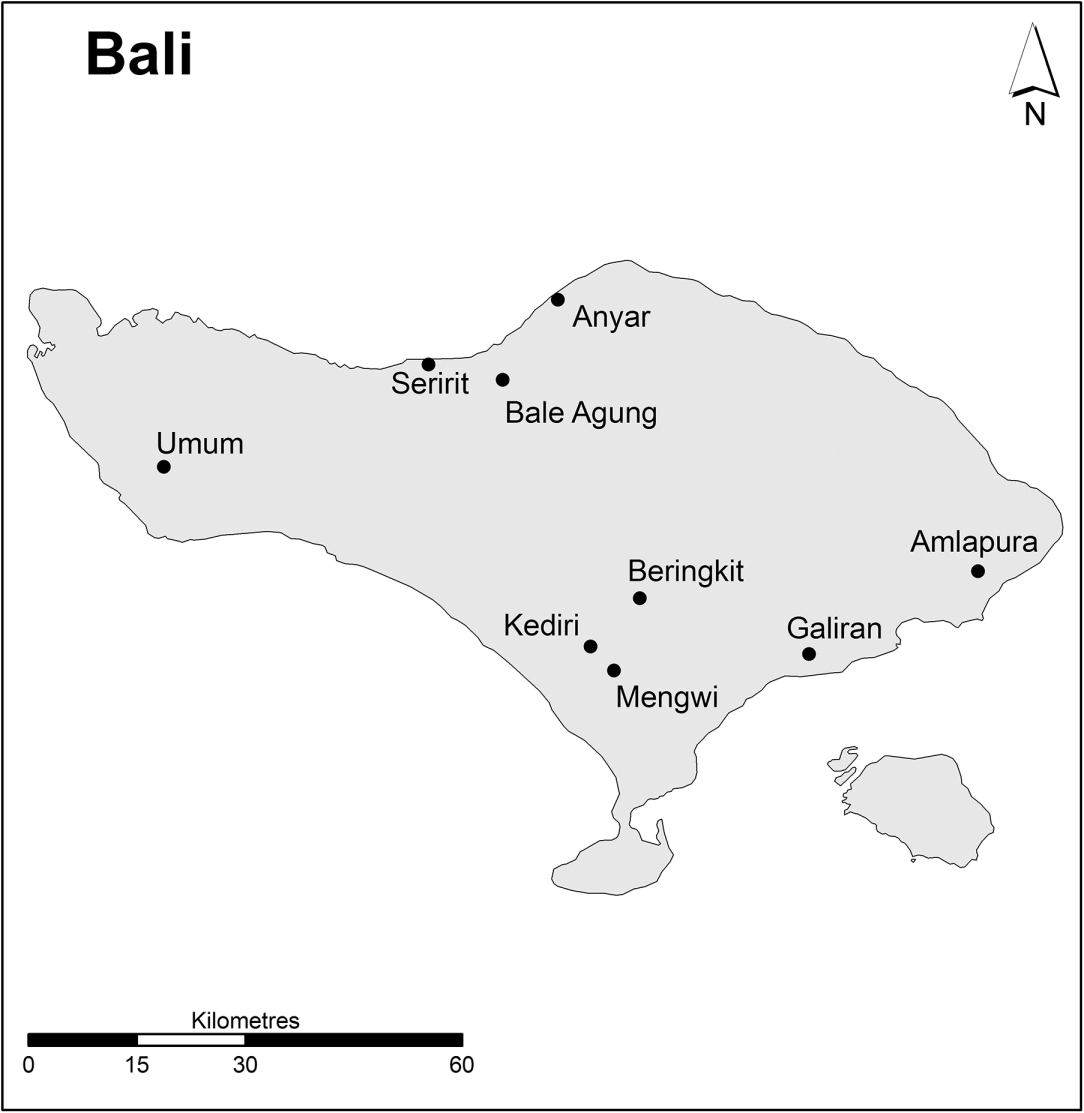
Location of markets in Bali. (Source: Charles Sturt University).

**Fig 3:**
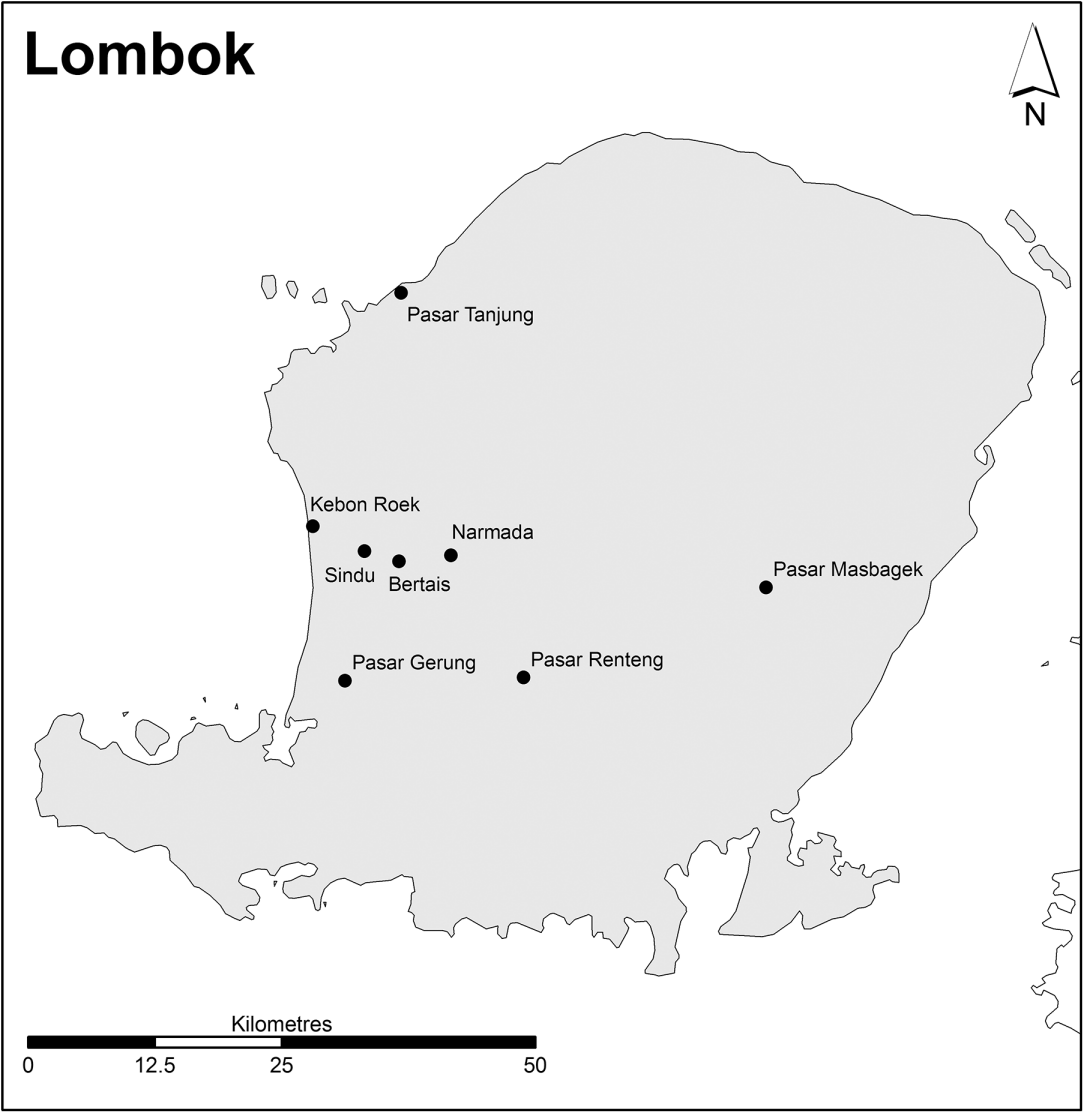
Location of markets in Lombok. (Source: Charles Sturt University).

At the commencement of the study it was estimated that the number of active live bird markets in Bali was 109 and 36 in Lombok (Disease Investigation Centre, Bali). Due to limited resources a total of nine markets in Bali (Fig 2) and eight markets in Lombok (Fig 3) were selected as the focus of this study. Markets were selected based on the following criteria: 1) size of the market (Large: >25 traders and sold multiple live poultry species and other livestock; Medium: 10–25 traders and a mixture of commodities sold in addition to an assortment of live poultry but no other livestock and; Small: also a mixed commodities market but on a smaller scale with few live poultry species and less than 10 traders selling birds); 2) approximate volume of poultry (High: >2000; Moderate: 500–2000; Low: <500); 3) medium to high flow of road traffic (expected to have a larger customer base) surrounding market; 4) operating frequency (i.e. daily trading or 1–3 times per week); 5) poultry farm density (High: >10 poultry farms within a 1km radius of market; Moderate: >10 poultry farms within a 1–5km radius and Low: >10 poultry farms more than 5km radius around market); and 6) whether there had been any locally confirmed reports (i.e. confirmed by the Regional Office of Livestock and Animal Health) of HPAI H5N1 outbreaks in poultry in the previous 12 months. Selection criteria categories for each of the surveyed markets are provided in S1 Table and S2 Table for Bali and Lombok, respectively.

### Respondents

Study participants consisted of individuals who were selling live poultry (i.e. not slaughtered or dressed birds) at one of the 17 markets on the day interviews were being conducted, were available to be interviewed on the day of the visit and who agreed to be interviewed. Respondents were classified either as a vendor or a collector based on the individual’s role in the market. Vendors (i.e. retailers) are defined as traders who primarily sell live poultry directly to customers at the market from temporary or permanent stalls. Collectors (i.e. wholesalers) are responsible for the collection of chickens and ducks from all sectors of the poultry industry and for transporting them to markets. The collector will either pick up poultry from farms on the way to a live bird market or will house birds for a period of time at a central village collection area, or at their home, after they have been picked up or after they have been delivered to that point by a farmer.

### Data collection

A semi-structured questionnaire was developed in English and translated into Bahasa Indonesia. Two local teams, from Udayana University (Bali) and Mataram University (Lombok), conducted the translation to ensure any differences in local dialects were accounted for. A committee meeting was held between team members to clarify the concepts, wording and administration of each question prior to translation. After translation, two questionnaires were piloted at a single market on both islands and modified accordingly.

Questionnaires consisted of three open-ended and three fixed alternative questions (S3 Table) that aimed to identify: 1) common sources of AI information; 2) knowledge of how AI is introduced into markets; 3) knowledge of measures on how to prevent poultry from becoming infected at markets; 4) perceptions towards reporting of sudden or suspicious bird deaths; 5) perceptions of the importance of biosecurity in markets and; 6) willingness to implement strategies to improve biosecurity in markets. Questionnaires also elicited details of respondent’s socio-demographic background (e.g. age, gender, education, religion, occupation and trading experience at the surveyed market) and the type and volume of poultry sold the previous day. Questions 5 and 6 were only added to the questionnaire after the second round of data collection and therefore only presented to respondents in the final round of interviews.

Questionnaires were administered at the markets using face-to-face interviews by two local teams of experienced enumerators from the Faculty of Agriculture, Udayana University, Bali and the Research Centre for Rural Development, University of Mataram, Lombok. These non-government affiliated enumerators were selected to conduct interviews due to concerns that respondents might be intimidated by government personnel and thus less willing to provide truthful responses, such as answering questions pertaining to illegal activities. Interviewers were instructed to enter markets and approach traders as convenient (i.e. respondents were not pre-selected) and request traders to be interviewed. Interviewers read the questions to respondents and recorded responses on the questionnaire. Respondents were free to speak at length for open-ended questions. A total of three rounds of interviews were conducted between May 2008 and February 2009, with each interview lasting approximately 15 to 20 minutes. The target sample size was determined by estimating the total number of traders working at each market, resulting in an overall sample size of 470 respondents, averaging 25 to 30 respondents per market. However, differences in the numbers of traders working in each of the surveyed markets meant it was not possible to reach the target sample size for each market. Therefore additional interviews were conducted in markets with larger numbers of traders in an effort to reach the target sample size. Follow-up interviews were not conducted, however during the course of the interview process a number (n = 57) of respondents were unintentionally interviewed twice, in which case data from both interviews was combined (or averaged for numerical data) to create single entries for each question per respondent. The total number of interviews conducted during the three rounds of data collection at each of the 17 surveyed markets is provided in S1 Table and S2 Table for Bali and Lombok, respectively.

During the course of the first two rounds of data collection, it became evident that the number of collectors and the time they spent in markets was minimal compared to vendors. Interviewers (particularly in Bali) also mentioned having difficulty in finding collectors that had not already been interviewed. Therefore, vendors became the main target respondents for the final round of interviews, especially in Bali.

Two additional questions were added to the questionnaire for the final round of interviews to ascertain information on trader perceptions toward biosecurity and willingness to implement changes to improve biosecurity within the markets. As the final round of interviews largely targeted vendors, the two additional questions were only presented to vendors. For the purposes of this study, biosecurity was defined as “a set of preventative practices aimed at reducing the potential for transmission and spread of disease causing organisms (specifically, avian influenza) onto and between sites, animals and humans” [37]. To ensure a uniform understanding among respondents, the term “biosecurity” was defined at the onset of the interview and again immediately prior to each question using the term. Responses for the question on perceptions of the importance of biosecurity were based on a Likert-type scale, with importance rated on a scale of one to five (1 = not important or unnecessary, 2 = low importance, 3 = medium importance, 4 = high importance and 5 = very important or crucial).

Upon completion of interviews respondents were remunerated for their time, which consisted of a monetary gift of 50,000IDR (approximately AUD5) for respondents in Bali and a t-shirt with an avian influenza awareness message for respondents in Lombok. Respondents were not informed prior to the interview that compensation or a gift would be provided to limit the possibility of bias with regard to participation and responses.

### Data Analysis

Data were translated to English and entered into a relational database (Microsoft Office Access 2007). Prior to analysis, respondents were further categorized based on the type of poultry sold (i.e. chickens, ducks or both) and on the volume of poultry sold per day—hobby (≤ 34 birds), small (35–89), medium (90–214) or large (≥215). “Chicken” refers to all varieties of *Gallus gallus domesticus* birds and “duck” refers to birds belonging to the *Anatidae* family, excluding geese and swans. Trader size categories were created using quartiles of the total volume of poultry sold the previous trading day as reported by each respondent.

Due to the small number of respondents with post-secondary school (i.e. college or university) education (n = 9), all of whom were from Bali, college, university and senior high school educated respondents were grouped together for analytical purposes. Trader knowledge was assessed by comparing the mean number of valid responses between groups for each question. Valid responses were defined as sources of transmission (for question on “how markets can become infected”) or preventative measures which fit with WHO or FAO avian influenza epidemiology and guidelines [37, 38].

Statistical analyses were conducted in R version 2.13.0 (R Core team, 2014). Categorical data was analyzed using Pearson’s chi-squared (χ^2^) test. In cases where cell counts were very small Pearson’s χ^2^ test with simulated p-value (based on 2000 replicates) was used. Continuous data was analyzed using the Spearman’s Rho test. Comparisons of two-level categorical and interval continuous data were analyzed using the t-test and where categorical data consisted of more than two levels, ANOVA was used. Post-hoc analysis of ANOVA results were assessed using the Tukey HSD test. A confidence interval of 95% was used as the measure of statistical significance in all analyses.

Multinomial logistic regression was used to analyze the influence of HPAI information sources on trader knowledge of HPAI transmission and prevention (response variables). Analysis of information sources (explanatory variables) used the count of responses provided by respondents or the type of source reported. However, for the latter, only television and personal communication were included in the models due to the low frequency at which other HPAI information sources were reported. Response variables consisted of the count data of responses for the questions pertaining to ‘how markets can become infected with HPAI’ or ‘measures to prevent HPAI in poultry at markets’. Logistic regression was performed using R version 2.13.0 (R Core team, 2014). Odds ratios were calculated for the effects of HPAI information sources on trader knowledge of HPAI transmission and prevention. A confidence interval of 95% and p-values were used as measures of statistical significance in all models.

## Results

### Socio-demographic background of respondents in Bali and Lombok

A total of 413 traders of similar proportions from Bali (47%, n = 195) and Lombok (53%, n = 218) participated in this study (Table 1). Refusal rate was zero. Most (89%) respondents were male with a mean age of 45 years (range 19–81) and had not completed junior high school (71%, n = 294). Nearly a third (32%, n = 62) of Balinese respondents had completed senior high school or post-secondary studies compared to only 6% (n = 12, p < .001) of Lombok respondents. There were significantly more female respondents interviewed in Bali (23%, n = 45) than in Lombok (1%, n = 2, p < .001).

**Table 1:** Socio-demographic background of poultry traders interviewed at live bird markets in Bali and Lombok during 2008–2009 stratified by location.

Demographic data		Location		P-value
		Bali (n = 195)	Lombok (n = 218)	
Gender				
	Male	150	216	<0.001
	Female	45	2	
Age (years)				
	Mean	45.0	46.6	0.143^a^
	Range	19–81	23–70	n/a
Education level achieved				
	No formal	14	99	<0.001
	Primary school	92	89	
	Junior high school	27	18	
	Senior high school (or post-secondary education)	62^b^	12	
Religion				
	Hindu	183	2	<0.001^c^
	Islam	12	216	
Trading experience (years)				
	Mean	15.5	14.1	0.178^d^
	Range	<1–50	1–48	n/a

^a^T-test (t = -1.47, df = 362, p-value: 0.143).

^b^Includes nine respondents with post-secondary school education.

^c^Simulated p-values based on 2000 replicates.

^d^T-test (t = 1.35, df = 347 p-value: 0.178).

Background profiles of respondents stratified by trader type (i.e. vendor or collector) and volume of poultry sold revealed significant differences in both education and gender. The highest proportion of female respondents was seen among large traders (21%, n = 18) and vendors (13%, n = 41). Education levels were typically highest among collectors and large traders and lowest among hobby-sized traders. There were no significant differences in the socio-demographic profiles of respondents selling only one species of poultry or both chickens and ducks, except that traders selling only ducks had slightly more poultry trading experience. Differences in demographic profiles of traders based on the type of trader, poultry species and volume of poultry sold are outlined in Table 2.

**Table 2:** Socio-demographic background of poultry traders interviewed at live bird markets in Bali and Lombok during 2008–2009 stratified by trader category.

Demographic data		Trader type^a^			Poultry type^b^				Trader size^c^				
		Vendor (n = 307)	Collector (n = 106)	p-value	Chicken (n = 283)	Duck (n = 58)	Chicken & duck (n = 72)	p-value	Hobby (n = 103)	Small (n = 94)	Medium (n = 112)	Large (n = 104)	p-value
Island													
	Bali	150	45	n/a	124	30	41	0.132	29	34	54	78	<0.001
	Lombok	157	61	n/a	159	28	31		74	60	58	26	
Gender													
	Female	41	6	0.048	27	7	13	0.144	9	4	16	18	0.019
	Male	266	100		256	51	59		94	90	96	86	
Age (years)													
	Mean	46.2	44.8	0.219^d^	45.3	47.9	46.2	0.254^e^	48.3	46.2	45.5	43.4	0.012^f^
	Range	19–81	23–70		19–81	25–70	23–74		24–80	23–77	19–81	20–74	
Education level achieved													
	No formal	85	28	0.020	79	17	17	0.416	36	33	31	13	<0.001
	Primary school	145	36		127	26	28		49	41	51	40	
	Junior high school	27	18		33	5	7		10	6	14	15	
	Senior high school	50	24		44	10	20		8	14	16	36	
Trading experience (years)													
	Mean	14.9	14.4	0.634^g^	14.2	17.8	14.8	0.053^h^	14.9	16.1	14.3	14.0	0.513^i^
	Range	1–50	0–48		1–48	0–48	1–50		1–43	1–50	1–48	0–48	

n/a: not assessed. Vendors were the primary target of the study.

^a^Category based on primary role of the respondent in the market at time of interview.

^b^Category based on the whether respondent sold chickens, ducks or both species on their previous day of trading.

^c^Category based on the volume of birds sold on the respondent’s previous day of trading.

^d^T-test (t = -1.24, df = 211, p-value: 0.219).

^e^One-way ANOVA (F_2,410_ = 1.37, p-value: 0.254).

^f^One-way ANOVA (F_3,409_ = 3.68, p-value: 0.012).

^g^T-test (t = -0.48, df = 188, p-value: 0.634).

^h^One-way ANOVA (F_2,410_ = 2.96, p-value: 0.053). Post-hoc analysis using Tukey HSD revealed a significant difference between chicken traders and duck traders (p-value: 0.049).

^i^One-way ANOVA (F_3,409_ = 0.77, p-value: 0.513).

### Sources of information on HPAI

Information on HPAI was derived from a variety of sources but the vast majority of traders (78%, n = 324) had learnt about the disease (and outbreaks occurring in Bali and Java) through television. Personal communication was also found to play an important role in information transfer, with approximately one third (n = 133) of all respondents obtaining information on HPAI from other people. Female traders (60%, n = 28) and participants in Bali (43%, n = 83) relied heavily on personal communication compared to male traders (29%, n = 105) and Lombok respondents (23%, n = 50) and these differences were highly significant (p < .001). Personal communication was also common among duck traders (43%, n = 25) although not significantly more than among chicken traders (29%, n = 81) or traders selling both species (38%, n = 27, p = 0.078).

In instances where traders relied on personal communication for HPAI information, it typically came from friends or local government officials, such as the village head and government livestock officers (especially for Bali markets). One vendor in Lombok mentioned learning about AI during a meeting on the disease.

Nearly 10% (n = 38) of respondents had never been exposed to information on AI. Uneducated traders (18%, n = 20, p_simulated_ < .001) and traders selling ducks (17%, n = 10, p = .035) were twice as likely to have had no exposure to HPAI information compared to most other traders. The maximum number of information sources reported by any single respondent was four but, the overall mean was relatively low at only 1.3. There was no significant difference between male and female respondents (p>0.05).

Significant differences were identified in the mean number of information sources reported by respondents with different education status (p < .001). The highest educated respondents (i.e. senior school, tertiary or university) accessed information from the greatest number of sources (Mean = 1.7) and uneducated respondents from the least (Mean = 1.0). Junior educated respondents reported more information sources (Mean = 1.5) than uneducated and more than primary educated respondents (Mean = 1.2). Post-hoc analysis revealed that differences between senior and uneducated respondents and senior and primary educated respondents were highly significant (p < .001). Differences were also significant between uneducated and junior educated respondents but not between respondents of other education categories (p>0.05). Differences were also identified in mean number of sources between locations, trader types and trader sizes but not between traders categorized by poultry type (Table 3).

**Table 3:** HPAI information sources reported by poultry traders interviewed at live bird markets in Bali and Lombok during 2008–2009, stratified by trader category.

Types of information sources	Number (%) of respondents														
	Location			Trader type			Poultry type trader				Trader size				
	Bali (n = 195)	Lombok (n = 218)	p-value	Vendor (n = 307)	Collector (n = 106)	p-value	Chicken (n = 283)	Duck (n = 58)	Chicken & duck (n = 72)	p-value	Hobby (n = 103)	Small (n = 94)	Medium (n = 112)	Large (n = 104)	p-value
Television	159 (81.5)	165 (75.7)	0.186	236 (76.9)	88 (83.0)	0.234	229 (80.9)	35 (60.3)	60 (83.3)	0.001	71 (68.9)	82 (87.2)	88 (78.6)	83 (79.8)	0.019
Personal communication	83 (42.6)	50 (22.9)	<0.001	93 (30.3)	40 (37.7)	0.196	81 (28.6)	25 (43.1)	27 (37.5)	0.078	26 (25.4)	28 (29.8)	39 (34.8)	40 (38.5)	0.191
Radio	28 (14.4)	2 (0.9)	<0.001	23 (7.5)	7 (6.6)	0.931	24 (8.5)	1 (1.7)	5 (6.9)	0.201^a^	5 (4.9)	9 (9.6)	5 (4.5)	11 (10.6)	0.201
Print (e.g. newspaper)	31 (15.9)	1 (0.5)	<0.001	22 (7.2)	10 (9.4)	0.588	21 (7.4)	5 (8.6)	6 (8.3)	0.926^a^	5 (4.9)	6 (6.4)	5 (4.5)	16 (15.4)	0.009
Visual print (e.g. poster/pamphlet	6 (3.1)	6 (2.8)	1.000	6 (2.0)	6 (5.7)	0.085^a^	5 (1.8)	2 (3.4)	5 (6.9)	0.059^a^	1 (1.0)	3 (3.2)	3 (2.7)	5 (4.8)	0.472^a^
No exposure to HPAI information	16 (8.2)	22 (10.1)	0.623	32 (10.4)	6 (5.7)	0.205^a^	25 (8.8)	10 (17.2)	3 (4.2)	0.035	13 (12.6)	3 (3.2)	12 (10.7)	10 (9.6)	0.120
Mean no. of sources reported per respondent	1.6	1.0	<0.001^b^	1.2	1.4	0.024^c^	1.3	1.2	1.4	0.169^d^	1.1	1.4	1.2	1.5	<0.001^e^

^a^Simulated p-values based on 2000 replicates.

^b^T-test (t = 7.42, df = 296, p-value: <0.001).

^c^One-way ANOVA (F_1,411_ = 5.10, p-value: 0.024).

^d^One-way ANOVA (F_2,410_ = 1.79, p-value: 0.169).

^e^One-way ANOVA (F_3,409_ = 6.44, p-value: <0.001). Post-hoc analyses revealed significant differences were only evident between large and hobby traders (p < .001) and hobby and small-sized traders (p = 0.024).

### Knowledge of how HPAI is introduced to the poultry market

The most common potential source of contamination reported by traders was “infected poultry”, which was reported by more than half (n = 238) of all respondents. Knowledge of other common pathways for HPAI introduction was limited in comparison (Table 4). Large traders were slightly more aware of the potential of fomites such as vehicles and cages as sources of contamination than other trader sizes (Table 4). Among poultry type traders, a higher proportion of respondents selling both chickens and ducks were aware that poultry infected with HPAI can potentially contaminate markets compared to traders selling only one of these species (72% vs. 56% of chicken traders and 45% of duck traders, p = 0.006). Similarly, collectors also demonstrated a greater awareness compared to vendors based on the number of potential pathways for HPAI introduction they were able to correctly identify (Mean = 1.1 vs. 0.6, p < .001) and the low proportion of respondents that had no knowledge (24% vs. 47%, p < .001).

**Table 4:** HPAI transmission routes reported by poultry traders interviewed at live bird markets in Bali and Lombok during 2008–2009, stratified by trader category.

Pathways for HPAI introduction into LBMs	Number (%) of respondents														
	Location			Trader type			Poultry type trader				Trader size				
	Bali (n = 195)	Lombok (n = 218)	p-value	Vendor (n = 307)	Collector (n = 106)	p-value	Chicken (n = 283)	Duck (n = 58)	Chicken & duck (n = 72)	p-value	Hobby (n = 103)	Small (n = 94)	Medium (n = 112)	Large (n = 104)	p-value
Infected poultry	106 (54.3)	132 (60.6)	0.241	161 (52.4)	77 (72.6)	< .001	160 (56.5)	26 (44.8)	52 (72.2)	0.006	56 (54.4)	58 (61.7)	60 (53.6)	64 (61.5)	0.476^a^
Infected wild birds	5 (2.6)	3 (1.4)	0.485^a^	4 (1.3)	4 (3.8)	0.217^a^	4 (1.4)	1 (1.7)	3 (4.2)	0.280^a^	0	1 (1.1)	2 (1.8)	5 (4.8)	0.068^a^
Contaminated vehicles	17 (8.7)	0	<0.001	9 (2.9)	8 (7.5)	0.056^a^	8 (2.8)	3 (5.2)	6 (8.3)	0.079^a^	0	2 (2.1)	2 (1.8)	13 (12.5)	<0.001^a^
Contaminated cages	23 (11.8)	22 (10.1)	0.692	24 (9.5)	21 (19.8)	0.001	30 (10.6)	4 (6.9)	11 (15.3)	0.300	6 (5.8)	10 (10.6)	8 (7.1)	21 (20.2)	0.003
Contaminated clothing or footwear	3 (1.5)	0	0.102^a^	0	3 (2.8)	0.015^a^	0	2 (3.4)	1 (1.4)	0.015^a^	0	0	0	3 (2.9)	0.039^a^
‘Do not know’	83 (42.6)	85 (39.0)	0.524	143 (46.6)	25 (23.6)	<0.001	119 (42.0)	32 (55.2)	17 (23.6)	<0.001	47 (45.6)	35 (37.2)	51 (45.5)	35 (33.7)	0.193
Mean no. of responses per respondent	0.8	0.7	0.332^b^	0.6	1.1	<0.001^c^	0.7	0.6	1.0	0.003^d^	0.6	0.8	0.6	1.0	<0.001^e^

^a^Simulated p-values based on 2000 replicates.

^b^T-test (t = 0.97, df = 355, p-value: 0.332).

^c^T-test (t = 4.57, df = 152.11, p-value: <0.001).

^d^ANOVA(F_2,404_ = 5.79, p-value: 0.003). Post-hoc analysis revealed significant differences between ‘chicken and duck’ and ‘duck’ traders (p = 0.007) and between ‘chicken and duck’ and ‘chicken’ traders (p = 0.007).

^e^ANOVA(F_3,409_ = 6.86, p-value: <0.001). Post-hoc analysis revealed significant differences between large and hobby-sized traders (p<0.001) and large and medium-sized traders (p = 0.001.

Most traders were unable to identify more than one possible source of contamination (Mean = 0.76) and only eight traders provided three or more sources (maximum of five) and 41% (n = 168) had no knowledge of how HPAI is transmitted. This was particularly evident among duck traders who had the highest proportions of respondents that were unable to identify a source of contamination (55%, n = 32). Education was strongly associated with trader ability to identify potential HPAI introduction pathways (F_3,409_ = 4.57, p = 0.004). Post-hoc analysis revealed that respondents with senior education or higher were able to provide 30% more responses than primary educated (Estimate = 0.31, p = 0.017), and 40% more than uneducated respondents (Estimate = 0. 36, p = 0.008). There was no significant difference in mean number of responses (i.e. potential HPAI introduction pathways) between male and female traders (Mean = 0.7 vs. 0.9, p>0.05).

Sixteen (7%) respondents from Lombok seemed to have doubts about the presence of HPAI on their island (2%, n = 5), in the bird species they were selling (3%, n = 7), or whether HPAI exists at all (2%, n = 4). Respondents interviewed in Bali made no such comments.

### Knowledge of how to prevent HPAI transmission

Cleaning and disinfecting cages was recognized as the most important step in preventing HPAI in poultry at markets yet it was only reported by half of all respondents (n = 232) and 40% (n = 166) were aware that disposing of sick and dead birds minimizes the risk of virus transmission. Vaccination was not rated highly (17%, n = 71) as a method for preventing HPAI except among traders selling both chickens and ducks (28%, n = 20).

Traders interviewed in Bali appeared to have a better knowledge of good biosecurity practices than Lombok traders, with more than 80% (n = 157) able to list at least one preventative measure compared to only 57% (n = 123) of Lombok traders. Although the maximum number of preventative measures reported by respondents in both locations was very similar (6 vs. 5 for Bali and Lombok, respectively), Bali traders reported a greater variety of preventative measures on average than Lombok respondents (Mean = 2.3 vs 1.2, p < .001).

Of the two trader types, collectors were more knowledgeable of good biosecurity practices than vendors, particularly in their awareness of the importance of cleaning cages and vehicles, and separating different bird species (Table 5). More than 85% (n = 91) provided at least one preventative measure compared to 62% (n = 191) of vendors and the average number of preventative measures was also significantly greater (Mean = 2.1 vs. 1.6, p = 0.002). There was little difference in awareness of preventative measures among traders based on species or volume of birds sold, however traders selling both chickens and ducks, and large traders, did provide a greater average number of preventative measures compared to their peers.

**Table 5:** Preventative measures reported by poultry traders interviewed at live bird markets in Bali and Lombok during 2008–2009, stratified by trader category.

Preventative measures	Number (%) of respondents														
	Location			Trader type			Poultry type trader				Trader size				
	Bali (n = 195)	Lombok (n = 218)	p-value	Vendor (n = 307)	Collector (n = 106)	p-value	Chicken (n = 283)	Duck (n = 58)	Chicken & duck (n = 72)	p-value	Hobby (n = 103)	Small (n = 94)	Medium (n = 112)	Large (n = 104)	p-value
Vaccinate poultry	37 (19.0)	34 (15.6)	0.437	49 (16.0)	22 (20.8)	0.328	43 (15.2)	8 (13.8)	20 (27.8)	0.031	16 (15.5)	17 (18.1)	14 (12.5)	24 (23.1)	0.211
Clean cages	135 (69.2)	97 (44.5)	<0.001	156 (50.8)	76 (71.7)	<0.001	153 (54.1)	31 (53.4)	48 (66.7)	0.142	55 (53.4)	54 (57.4)	59 (52.7)	64 (61.5)	0.541
Clean stall area	81 (41.5)	35 (16.1)	<0.001	87 (28.3)	29 (27.4)	0.946	75 (26.5)	16 (27.6)	25 (34.7)	0.381	21 (20.4)	25 (26.6)	34 (30.4)	36 (34.6)	0.132
Clean vehicles	65 (33.3)	12 (5.5)	<0.001	47 (15.3)	30 (28.3)	0.005	49 (17.3)	14 (24.1)	14 (19.4)	0.469	13 (12.6)	11 (11.7)	21 (18.8)	32 (30.8)	0.001
Separate different bird species	30 (15.4)	16 (7.3)	0.015	28 (9.1)	18 (17.0)	0.041	26 (9.2)	9 (15.5)	11 (15.3)	0.177	9 (8.7)	6 (6.4)	15 (13.4)	16 (15.4)	0.157
Separate birds from different sources	5 (2.6)	3 (1.4)	0.489^a^	6 (2.0)	2 (1.9)	1.000^a^	6 (2.1)	0	2 (2.8)	0.591	1 (1.0)	3 (3.2)	0	4 (3.8)	0.129^a^
Dispose of sick and dead birds	102 (52.3)	64 (29.4)	<0.001	119 (38.8)	47 (44.3)	0.371	113 (39.9)	19 (32.8)	34 (47.2)	0.244	48 (46.6)	30 (31.9)	44 (39.3)	44 (42.3)	0.198
‘Do not know’	38 (19.5)	93 (42.7)	<0.001	116 (37.8)	15 (14.1)	<0.001	93 (32.9)	22 (37.9)	16 (22.2)	0.122	34 (33.0)	30 (31.9)	37 (33.0)	30 (28.8)	0.904
Mean no. of responses	2.3	1.2	<0.001^b^	1.6	2.1	0.002^c^	1.6	1.7	2.2	0.046^d^	1.6	1.6	1.7	2.1	0.038^e^

^a^Simulated p-values based on 2000 replicates.

^b^T-test (t = 7.78, df = 378.52).

^c^T-test (t = 3.17, df = 197.17).

^d^One-way ANOVA (F_2,410_ = 3.10, p-value: 0.046).

^e^One-way ANOVA (F_2,409_ = 2.86, p-value: 0.038).

Mean number of preventative measures identified by female traders was more than 1.5 times greater than male traders (Mean = 2.7 vs. 1.6, respectively, p < .001). However, to determine whether these differences were merely a reflection of the differences observed between locations (as mentioned above), the mean number of preventative measures reported by female and male traders in Bali (where the majority of female traders were interviewed) were compared. The findings indicate that despite a smaller difference in mean values, female traders had significantly better knowledge of preventative measure than male traders (Mean = 2.8 vs. 2.2, t = 2.31, df = 80.46, p = 0.02). Education was also associated with knowledge of preventative measures (F_3,409_ = 16.77, p < .001). Post-hoc analysis revealed that primary and junior educated respondents were able to provide 60% more responses (Estimate = 0.58, p = 0.005) and 100% (Estimate = 0.98, p<0.001) respectively, than uneducated respondents. Senior educated respondents were able to provide 150% (Estimate = 1.52, p < .001) more than uneducated respondents and 90% (Estimate = 0.93, p < .001) more than primary educated.

### Willingness to report suspected cases of HPAI

Of the 413 respondents, a total of 408 provided a response to the question pertaining to reporting of sudden or suspicious bird deaths (i.e. any number of unexplained sudden deaths of birds with no prior signs of ill health). The five that elected not to respond were all male respondents from Bali, consisting of three vendors and two collectors.

The trend among all traders was to not report suspicious bird deaths (Table 6), particularly among traders interviewed in Bali (74%, n = 140) or those with a primary school level of education (75% (n = 135). Traders who had completed junior school were more willing to report than traders who had completed senior school (Table 6). Furthermore, seven out of nine respondents who had completed post-secondary school studies (included as part of the senior-educated group) stated that they would not report. Female traders were also more reluctant to report with 83% (n = 39) saying no, compared to 66% (n = 238) of male traders, although the differences were not within significant levels (p = 0.058). There were no significant differences in reporting behavior between vendors and collectors, or between traders categorized on type of poultry or volume of poultry sold. Of 85 (21%) traders who provided details on whom they would inform, 76% (n = 85) said they would inform a village official, 12% (n = 10) would report to a government animal health worker, 5% (n = 4) to a market manager and 4% (n = 3) would tell a veterinarian. In addition, 4% (n = 3) said they would notify an officer but did not extrapolate further.

**Table 6:** Reporting of suspicious or sudden birds deaths as reported by poultry traders interviewed at live bird markets in Bali and Lombok during 2008–2009.

Factors		Number (%) of respondents			p- value
		Would report	Would not report	Possibly would report	
Location					
	Bali	31 (16.3)	140 (73.7)	19 (10.0)	0.046
	Lombok	56 (25.7)	137 (62.8)	25 (11.5)	
Gender					
	Male	81 (22.4)	238 (65.9)	42 (11.6)	0.058
	Female	6 (12.8)	39 (83.0)	2 (4.3)	
Education level achieved					
	No formal	25 (22.3)	75 (67.0)	12 (10.7)	0.022
	Primary school	27 (15.1)	135 (75.4)	17 (9.5)	
	Junior high school	17 (38.6)	22 (50.0)	5 (11.4)	
	Senior high school	18 (24.7)	45 (61.6)	10 (13.7)	
Trader type					
	Vendor	65 (21.4)	211 (69.4)	28 (9.2)	0.208
	Collector	22 (21.1)	66 (63.5)	16 (15.4)	
Poultry species					
	Chicken	63 (22.5)	188 (67.1)	29 (10.4)	0.655
	Duck	13 (22.8)	39 (68.4)	5 (8.8)	
	Chicken & duck	11 (15.5)	50 (70.4)	10 (14.1)	
Trader size					
	Hobby	26 (25.5)	71 (69.6)	5 (4.9)	0.192
	Small	17 (18.3)	61 (65.6)	15 (16.1)	
	Medium	22 (19.8)	79 (71.2)	10 (9.0)	
	Large	22 (21.6)	66 (64.7)	14 (13.7)	

In addition to the 87 traders who would report suspected HPAI cases, 44 (11%) traders also stated that they may ‘possibly report’. More than one third (n = 15) of the 44, who were all from Lombok, said they probably would report suspicious bird deaths if they knew where to report. Uncertainty of whether to report or not was also due to traders not having any previous experience with suspicious bird deaths (14%, n = 6) or because they believed it was unlikely to occur in their flocks (9%, n = 4). A further two (5%) traders said their decision to report would depend largely on the number of birds that died. However, neither of the two traders specified what number of bird deaths would need to occur in order for them to feel it deserves reporting.

Reasons for not wanting to report suspicious bird deaths were similar to those described above. Of the 137 Lombok traders who said they would not report, 58% (n = 79) were unaware of whom to inform and 18% (n = 25) it was too inconvenient due to the distance they had to travel to find someone to report to. Not having experienced suspicious bird deaths was also a reason for non-reporting in Lombok (12%, n = 16) and six (4%) preferred to handle the situation alone (no further details provided). Five (4%) traders believed that reporting was unnecessary because AI was not a problem in the type of birds they sold (ducks or chickens) or in Lombok and three (2%) traders were reluctant to report because of potential consequences such as “bringing shame”. The remaining three traders in Lombok who were unwilling to report did not specify a reason. In Bali, only 27 (19%) of 140 traders provided a reason for not wanting to report suspicious bird deaths and 93% (n = 25) of those said it was because they had not experienced suspicious bird deaths. One (0.7%) respondent was unaware of who deaths should be reported to and there was also one (0.7%) respondent who would prefer to handle the situation alone. No other traders from provided reasons for not wanting to report suspicious bird deaths.

### Perceptions on the importance of biosecurity in markets

During the final round of interviews respondents (n = 188) were asked to rate the importance of biosecurity in markets on a scale of one (not important) to five (very high). Scores ranged between one and four for Lombok and from one to five for Bali. Biosecurity within live bird markets was perceived to be more important by vendors in Bali (Mean = 3.9, IQR = 3.0–5.0) than in Lombok (Mean = 2.4, IQR = 2.0–4.0, p_sim_ < .001). Vendors with different education levels also perceived biosecurity differently. Uneducated vendors rated biosecurity to be of lower importance (Mean = 2.5, IQR = 2.0–3.0) than vendors with primary (Mean = 3.2, IQR = 2.0–4.0), junior (Mean = 3.3, IQR = 2.0–4.0) and senior school level education (Mean = 4.3, IQR = 4.0–5.0). Similarly, perceptions of biosecurity importance also appeared to increase with trader size categories. Large vendors had an average score of 3.7 (IQR = 2.0–5.0) compared to a mean of 3.0 for small and medium sized vendors (IQR = 2.0–4.0 and 2.0–3.0, respectively) and 2.7 (IQR = 2.0–4.0) for hobby-sized vendors. Differences in mean scores between education levels and trader sizes were found to be significant (p_sim_ = 0.002 and p_sim_ < .001, respectively). However, there was no significant difference in perceptions of biosecurity importance between traders selling chickens (Mean = 3.5, IQR = 2.0–4.0), ducks (Mean = 3.2, IQR = 2.0–4.0) or both species (Mean = 3.5, IQR = 2.2–4.0, p_sim_ >0.05).

### Willingness to implement strategies to improve biosecurity in markets

Vendors interviewed in the final round of data collection were also asked about whether they would be willing to implement strategies to improve biosecurity (in general terms rather than specific measures) within markets. Nearly half (n = 90) of the vendors said yes. Two and a half times more respondents in Bali were willing to implement changes to improve biosecurity in markets than in Lombok (n = 65 vs. 25 with yes responses. P<0.001). Respondents categorized as medium or large-sized or who had a minimum of a senior school education were also more willing to implement changes when compared to respondents selling smaller quantities of birds or who were less educated (Table 7).

**Table 7:** Willingness to implement measures to improve biosecurity in markets reported by 188 poultry traders interviewed at live bird markets in Bali and Lombok during final round of interviews in 2009.

Trader category		Number (%) of respondents			
		Willing	Not willing	Possibly	p-value
Location					
	Bali (n = 95)	65 (68.4)	19 (20.0)	11 (11.6)	<0.001
	Lombok (n = 93)	25 (26.9)	33 (35.5)	35 (37.6)	
Education					
	None (n = 48)	12 (8.3)	22 (45.8)	14 (29.2)	0.009^a^
	Primary (n = 99)	55 (55.6)	22 (22.2)	22 (22.2)	
	Junior (n = 19)	9 (47.4)	4 (21.1)	6 (31.6)	
	Senior (n = 22)	14 (63.6)	4 (18.2)	4 (18.2)	
Poultry species					
	Chicken (n = 117)	59 (50.4)	31 (26.5)	27 (23.1)	0.183
	Duck (n = 30)	14 (46.7)	5 (16.7)	11 (36.7)	
	Chicken & duck (n = 38)	16 (42.1)	15 (39.5)	7 (18.4)	
Trader size					
	Hobby (n = 44)	18 (40.9)	13 (29.5)	13 (29.5)	0.007
	Small (n = 31)	10 (32.3)	17 (54.8)	4 (12.9)	
	Medium (n = 54)	31 (57.4)	8 (14.8)	15 (27.8)	
	Large (n = 59)	31 (52.5)	14 (23.7)	14 (23.7)	

^a^Simulated p-value.

More than one-third (n = 33) of vendors in Lombok were against implementing changes, compared to 20% (n = 19) in Bali. Only three (16%) of the 19 respondents in Bali provided a reason for why they would not be willing to implement measures to improve biosecurity within markets. Two of the three said they felt there was no problem and therefore no interventions were required, and the third respondent was unwilling due to inconvenience. In contrast, 31 (94%) of 33 vendors in Lombok provided a reason for their unwillingness to improve biosecurity in markets. The majority (55%, n = 17) was uncertain of what would be involved (e.g. cost, time, etc), which made them reluctant to be open to implementing improvements. One third (n = 11) of the respondents in Lombok who provided a reason felt that the responsibility of improving biosecurity belonged to market managers or animal health officers rather than themselves, particularly vendors selling smaller quantities of birds. A further two (6%) respondents felt that it was unnecessary to improve biosecurity in markets because they believed that HPAI was not an issue in Lombok. One respondent was unwilling due to concerns about the type of measures that would be implemented and how this would impact on their sales. The example given by the respondent was the use of face masks, which had been recommended to him previously (no mention of by whom) that he felt was uncomfortable and he feared that it might deter customers.

In addition to respondents that were either for or against making changes, there were also 46 (24%) respondents, mostly from Lombok (76%, n = 35), who said they might be willing to implement strategies to improve biosecurity in markets. Respondents from Bali gave no explanations for their uncertainty. Among Lombok respondents, willingness largely depended on whether financial assistance and training would be provided from the local government or animal health officers (60%, n = 21) or if the measures would improve the health of their birds (n = 6, 17%). There were also respondents who would only be willing to implement changes if all traders at the market equally participated (9%, n = 3) or if the measures were not too inconvenient (the only example provided specifically mentioned wearing face masks as an example of inconvenient).

### Influence of information sources on trader knowledge of HPAI transmission and prevention

Analysis of information sources and trader knowledge using multinomial logistic regression revealed a positive and statistically significant association between the number of information sources reported by respondents and ability to identify HPAI transmission routes and knowledge of preventative measures. These results indicate that for every additional source of information a trader is exposed to we would expect to see a 50% increase the number of HPAI transmission routes (OR 1.45, 95% CI: 1.27–1.65, p < .001) and preventative measures identified (OR 1.53, 95% CI: 1.40–1.66, p < .001). Sourcing information by personal communication would be expected to increase the likelihood of a respondent being able to identify HPAI transmission pathways by 35% (OR 1.35, 95% CI: 1.07–1.69, p = 0.009) and preventative measures by nearly 50% (OR 1.47, 95% CI: 1.27–1.71, p < .001) compared to respondents that do not receive information from personal communication. However accessing information from television would increase a trader’s chance of identifying HPAI preventative measures by 120% (OR 2.19, 95% CI: 1.75–2.79, p < .001) and HPAI transmission pathways by 130% (OR 2.32, 95% CI: 1.65–3.39, p < .001) compared to respondent that did not source information from television.

## Discussion and Conclusions

The overall low level of poultry trader knowledge about HPAI transmission and prevention found in this study, and reluctance to report suspected HPAI cases is concerning and may be a contributing factor to the country’s limited success in controlling and preventing the spread of the disease in poultry.

Other than infected birds, there was limited knowledge of the potential of fomites, including vehicles, clothing and footwear, except amongst some collectors and large traders. Furthermore, 40% of all poultry traders were unable to identify at least one HPAI transmission pathway despite the fact that 90% of respondents had been exposed to information on avian influenza. Studies conducted in other developing countries also reported lack of knowledge about AI amongst poultry traders and workers despite exposure to several sources of AI information [24, 26, 39].

In contrast, there was a broader knowledge of how to prevent HPAI transmission amongst respondents, particularly in Bali where there has been greater government intervention following outbreaks and more effective communication between public health and animal health agencies at the sub district and village level [40]. Vaccination of poultry was not considered to be necessary in the prevention of HPAI, except among some traders selling both chickens and ducks. Less emphasis has been placed on vaccination as a HPAI control measure in Bali and Lombok compared to other control measures such as culling [32], which may explain why few traders mentioned vaccination as a preventative measure. The greater knowledge of preventative measures among traders compared to understanding of HPAI transmission suggests that traders have been exposed to appropriate biosecurity practices or have gained knowledge of how to prevent disease in their poultry through experience but have limited exposures to how HPAI is transmitted or spread.

Education level appeared to be a major factor in the level of respondents’ knowledge on HPAI and biosecurity, as confirmed by numerous other studies [14, 21, 23–26]. This may explain why collectors and large traders, who were better educated, also had higher levels of knowledge than other trader categories. However it does not explain why knowledge of HPAI and biosecurity were higher among traders selling both chickens and ducks compared to traders selling only one species, considering education levels were not significantly different. The number and type of information sources were also not significantly different between traders based on poultry type. Therefore, it is difficult to interpret the differences in knowledge between traders based on poultry species from this study alone and possibly warrants further investigation.

A particularly interesting finding from our study was that being educated and having knowledge of HPAI transmission and prevention does not lead to better reporting behavior. Respondents who had completed senior school or post-secondary studies were no more likely to report suspicious or sudden bird deaths than uneducated traders, which correspond to findings from other studies that indicate education and knowledge do not always translate into adopting consistent recommended protective measures [23,24, 25, 31]. The low levels of reporting demonstrated in our study may be due to the frequent occurrence of poultry deaths in Indonesia meaning that poultry mortalities are considered normal and it is therefore difficult for traders or farmers to determine if illnesses or deaths are due to HPAI or something else [2, 29]. Fear of authority, possible penalties and lack of compensation can also influence people’s willingness to report suspected HPAI cases [26, 41]. The fact that traders in Bali were less willing to report than in Lombok may also be an indication that Bali traders have experienced negative consequences from reporting in the past. Reluctance and uncertainty surrounding reporting in Lombok was largely a result of traders not knowing where to report suggesting that overall reporting behaviour of traders in Lombok could be improved by if this information was provided.

Another interesting difference between Bali and Lombok identified in this study is trader perceptions toward biosecurity. Although the final two questions were presented only to vendors our findings demonstrate that vendors in Bali view biosecurity to be more important than in Lombok. The higher importance Bali traders place on biosecurity and greater willingness to take steps to improve biosecurity in markets could be the result of being more informed about preventative measures compared to Lombok traders. However it could also be because Lombok traders do not see HPAI to be a problem on their island or in the species of birds they sell. Perceptions toward biosecurity and willingness to make changes were also found to be more positive among traders with better education levels and traders selling larger quantities of birds. Considering that large traders were better educated may be one possible explanation for their more positive attitude toward biosecurity. However it is also possible that traders selling larger quantities of birds have more at stake and therefore place more emphasis on disease preventative measures than traders selling small quantities. The study could have been improved by asking respondents follow-up questions about what type of interventions they would be willing to adopt as it appears as though reluctance of some vendors was due to not knowing what would be involved.

As Naysmith [8] and Goodwyn et al. [42] emphasised, people have to actually believe that there is a significant threat to birds and humans to see a genuine reason to change (evident by the differences between Bali and Lombok respondents). Perceptions towards the efficacy of prevention measures have also been shown to influence adoption rates [43]. Therefore, recommended preventative strategies have to consider the many subtle social, economic and cultural perceptions of HPAI risk and people’s ability to change behaviour [2, 28]. For example, Naysmith’s [8] reporting of Bali poultry trader reluctance to wear face masks and gloves for fear that their customers will think that they or their birds are unhealthy is highly rational from a business perspective. Likewise, the belief that birds from Java were the only source of infection, not Bali chickens was based on cultural divisions, and was reinforced by government regulation to disinfect only selling areas occupied by Javanese traders [8].

Working with such social, economic and cultural realities requires a multi-faceted and multi-sectoral approach over a long period of time to lead to sustained implementation of preventive measures. A combination of regulation, education and economic incentives is needed. Regulatory measures such as rules, surveillance and penalties have been more effective when developed in consultation with traders and farmers [26, 28, 44]. As Alders et al. [2] reported, strict regulation of live bird markets without adequate consultation with traders can result in parallel trading of birds in ad hoc markets, which can contribute to further spread of the disease. Compensation for culled birds can also reduce the perceived disadvantages of reporting sick or dead birds [40] but the price has to match market price and be equitable and sustainable [2]. Regulatory measures are also more likely to be adopted if live bird market infrastructure is improved at the same time as regulations are introduced [44].

Engaging poultry traders in educational activities with health authorities could help to build relationships and trust [30, 41, 44]. Designing training courses for traders would provide opportunities to share information and experiences. Given the mix of ages, gender and education levels encountered amongst Bali and Lombok traders, and the differences in their knowledge of HPAI and biosecurity, it would be advisable to run training courses in peer groups so that the more educated and more HPAI experienced traders can share their knowledge and experiences with others. Education and training needs to focus on reinforcing existing practices in Bali and on introducing new practices in Lombok. This would help to facilitate the flow of practical and credible information by word of mouth from trader to trader, instead of relying on media messages alone, which may be misunderstood if too technical (e.g. using too many scientific terms) or could be ignored if too alarmist. Delivering messages via intermediaries (e.g. village heads, local farmers and traders) can also be more effective than mass media or government communication programs [29]. However, findings from this study demonstrated that television might be a highly effective medium for educating traders and improving awareness of HPAI. Furthermore, our findings also indicate that exposure to a variety of different mediums of information can also assist in improving trader knowledge of HPAI transmission and prevention. This may further explain why Bali respondents, collectors and traders selling large volumes of poultry, who all had greater access to HPAI information, appeared to have a better understanding of how HPAI can enter markets and of how to prevent the disease.

Several key points need to be considered when interpreting the findings of this study. Firstly, the focus of our study was on live bird markets and the majority of traders selling poultry in each of the surveyed markets were vendors meaning the two trader types were disproportionately represented, which may introduce a certain level of bias. However the high response rate enabled us to make statistically significant inferences between the two trader types. Markets were also represented disproportionately, mostly due to differences in the number of traders working at each site, and this meant that samples were too low to conduct statistical analyses to compare individual markets. Although respondents were not informed of compensation gifts prior to interviews, it is possible that participants learned of this from previous respondents and this may also have resulted in a certain level of bias in responses. It is also possible that bias was introduced into the study by assuming that respondents were aware of the existence of HPAI as a disease of poultry and that it can also cause disease in humans. Another important consideration when interpreting our findings is the possibility that respondents did not list every single source of information or every transmission pathway or preventative measure. Therefore it is possible that traders have had higher levels of exposure to information and better knowledge than our findings demonstrate. The small number of questions and absence of follow-up questions also limits the amount of information retrieved by the study, although our study revealed a number of interesting findings that would benefit from further investigation, as mentioned earlier.

In conclusion, findings from this study indicate that traders have varying degrees of knowledge and perceptions toward HPAI and biosecurity, which are largely dependent on their education and exposure to AI information. Biosecurity was perceived to be of greater importance by Balinese vendors who were also more willing to implement positive steps to improve biosecurity in market. Further investigation is needed to better understand the differences in knowledge and perceptions between the different trader categories. Given the low level of HPAI knowledge greater efforts are needed by multidisciplinary teams to engage local government, market managers and traders in the development of education programs, regulatory measures and incentive mechanisms. This will help ensure messages are appropriate and easily understood by people of all backgrounds and education levels.

## Supporting Information

S1 Table Total number of interviews conducted at each of the nine markets in Bali during each round of data collection. (DOCX)

S2 Table Total number of interviews conducted at each of the eight markets in Lombok during each round of data collection. (DOCX)

S3 Table Questions on HPAI knowledge and perceptions towards biosecurity. (DOCX)
