# Supplementary material for: Knowledge and Perceptions of Highly Pathogenic Avian Influenza (HPAI) among Poultry Traders in Live Bird Markets in Bali and Lombok, Indonesia
Source: PLoS One. 2015 Oct 2;10(10):e0139917. doi: 10.1371/journal.pone.0139917 (PMC4592001; doi:10.1371/journal.pone.0139917)
Supplement: S2 Table — (DOCX) [file pone.0139917.s002.docx]

S2 Table. Total number of interviews conducted at each of the nine markets in Lombok during each round of data collection.

| Market | Selection criteria category^a^ | Number of respondents | | | | | | |
| --- | --- | --- | --- | --- | --- | --- | --- | --- |
|  |  | Round 1 | | Round 2 | | Round 3 | |  |
|  |  | Vendors | Collectors | Vendors | Collectors | Vendors | Collectors | Total |
| Bertais | 1.1, 2.1, 3.1, 4.1, 5.1, 6.1 | 4 | 4 | 4 | 4 | 1 | 2 | 19 |
| Gerung | 1.1, 2.1, 3.1, 4.1, 5.1, 6.1 | 6 | 4 | 4 | 4 | 14 | 0 | 32 |
| Kebon Roek | 1.2, 2.1, 3.2, 4.1, 5.2, 6.1 | 6 | 2 | 6 | 2 | 2 | 0 | 18 |
| Masbagik | 1.1, 2.1, 3.1, 4.1, 5.1, 6.1 | 4 | 4 | 4 | 4 | 13 | 2 | 31 |
| Narmada | 1.3, 2.1, 3.2, 4.1, 5.1, 6.1 | 5 | 8 | 4 | 5 | 22 | 2 | 46 |
| Renteng | 1.1, 2.1, 3.1, 4.1, 5.1, 6.1 | 9 | 4 | 6 | 2 | 25 | 13 | 59 |
| Sindu | 1.2, 2.1, 3.2, 4.1, 5.2, 6.1 | 4 | 4 | 6 | 2 | 12 | 3 | 31 |
| Tanjung | 1.3, 2.1, 3.3, 4.1, 5.3, 6.1 | 4 | 4 | 5 | 4 | 4 | 0 | 21 |
| **Total** |  | **42** | **34** | **39** | **27** | **93** | **22** | **257** |

^a^Criteria categories:

1. Volume of birds (approximate only): >1000 (1.1); 200-1000 (1.2) and; <200 (1.3).

2. Traffic density: heavy (2.1); moderate (2.2) and; low (2.3).

3. Size of market: large (3.1); medium (3.2) and; small (3.3).

4. Trading frequency: daily (4.1) and; not daily (4.2).

5. Poultry farm density: high (5.1); moderate (5.2) and; low (5.3).

6. Poultry outbreaks of HPAI H5N1 reported: true (6.1) and; false (6.2).
