# Supplementary material for: Knowledge and Perceptions of Highly Pathogenic Avian Influenza (HPAI) among Poultry Traders in Live Bird Markets in Bali and Lombok, Indonesia
Source: PLoS One. 2015 Oct 2;10(10):e0139917. doi: 10.1371/journal.pone.0139917 (PMC4592001; doi:10.1371/journal.pone.0139917)
Supplement: S3 Table — (DOCX) [file pone.0139917.s003.docx]

S3 Table. Questions on HPAI knowledge and perceptions towards biosecurity

| HPAI knowledge and biosecurity perceptions questions | | Type of question | Response options |
| --- | --- | --- | --- |
| 1. Where did you learn about avian influenza (AI)? |  | Open-ended^a^ |  |
| 2. To your knowledge, how is AI introduced into live bird markets (LBMs)? |  | Open-ended^a^ |  |
| 3. What do you see as necessary to prevent or control AI in poultry at LBMs? |  | Open-ended^a^ |  |
| 4. Would you report suspicious or sudden deaths in birds? |  | Fixed-alternative | Yes, no, possibly |
|  | 4a. If yes, to whom would you report? | Open-ended |  |
|  | 4b. If no, why not? | Open-ended |  |
|  | 4c. If possibly, please explain. | Open-ended |  |
| *5. How important do you think biosecurity in LBMs is on a scale of 1 to 5? |  | Fixed-alternative | 1 (not important)  2 (low)  3 (moderate)  4 (high)  5 (very high) |
| *6. Would you be willing to implement strategies to improve biosecurity in LBMs? |  | Fixed-alternative | Yes, no, possibly |
|  | 6a. If not, why not? | Open-ended |  |
|  | 6b. If possibly, please explain. | Open-ended |  |

*Included in the final round of interviews only

^a^A list of expected responses were included in the questionnaire for interviewers to mark if reported by respondent. A space was also provided to include unlisted responses.
